# Valorization of Date By-Products: Enhancement of Antioxidant and Antimicrobial Potentials through Fermentation

**DOI:** 10.3390/antiox13091102

**Published:** 2024-09-12

**Authors:** Azin Khosravi, Seyed Hadi Razavi, Ines Castangia, Maria Letizia Manca

**Affiliations:** 1 Bioprocess Engineering Laboratory (BPEL), Department of Food Science, Engineering and Technology, Faculty of Agricultural Engineering and Technology, University of Tehran, Karaj 31587-77871, Iran; azinkhosravi@ut.ac.ir; 2 Department of Life and Environmental Sciences, University of Cagliari, University Campus, S.P. Monserrato-Sestu Km 0.700, 09042 Monserrato, Italy; mlmanca@unica.it

**Keywords:** solid-state fermentation, *Lactiplantibacillus plantarum*, *Limosilactobacillus reuteri*, phenolic profile, date by-products

## Abstract

The by-products from three varieties of dates—Mozafati, Sayer, and Kabkab—were subjected to solid-state fermentation using *Aspergillus niger* alone or in co-culture with *Lactiplantibacillus plantarum* or *Limosilactobacillus reuteri* to enhance their phenolic and flavonoid content, along with antioxidant and antimicrobial activities. Solid-state fermentation, being environmentally friendly and cost-effective, is particularly suitable for agricultural residues. Significant increases (*p* < 0.05) in total polyphenol content (TPC), total flavonoid content (TFC), and antioxidant power were observed post-fermentation, especially under co-culture conditions. The highest TPC (12.98 ± 0.29 mg GA/g) and TFC (1.83 ± 0.07 mg QE/g) were recorded in the co-culture fermentation of by-products from the Mozafati and Sayer varieties, respectively. HPLC analysis revealed changes in polyphenol profiles post-fermentation, with reductions in gallic and ferulic acids and increases in caffeic acid, p-coumaric acid, rutin, quercetin, and kaempferol. FT-IR analysis confirmed significant alterations in polyphenolic functional groups. Enhanced antimicrobial activity was also observed, with inhibition zones ranging from 8.26 ± 0.06 mm for Kabkab to 17.73 ± 0.09 mm for Mozafati. These results suggest that co-culture solid-state fermentation is a promising strategy for valorizing date by-products, with potential applications in nutraceuticals and/or pharmaceutical products and as valuable additives in the food industry.

## 1. Introduction

In recent years, the importance of nutrition has garnered increased attention, especially in regard to a healthier perspective for humans [1]. This growing awareness has underscored the importance of dietary choices, with an increasing emphasis on consuming diets rich in fruits and vegetables due to their vital role in mitigating the risk of chronic diseases [2]. These health benefits are largely attributed to the presence of bioactive compounds in plant-based foods, which assist in combating oxidative stress by maintaining a crucial balance between free radicals and antioxidants, thereby promoting overall well-being. Among these bioactive compounds, polyphenols are particularly important and are found in all types of plants, where they are produced as a defense against infections, oxidative stress, UV radiation, and predator attacks [3]. With their aromatic rings and hydroxyl groups, polyphenols act as natural antioxidants, whose activity has been linked to various therapeutic effects on the human body, including anti-carcinogenic, anti-inflammatory, antimicrobial, anti-thrombotic, and cardio-protective properties [4]. The date palm (*Phoenix dactylifera*), one of the oldest plants cultivated by humans, is widely distributed around the world, particularly in the Middle East and North Africa [5]. Today, date palms are consumed globally and are among the most important economic crops. Dates are an excellent source of simple carbohydrates, primarily glucose and fructose, which constitute 65–80% of their composition. They are also notable for their dietary fiber content (6.4–11.5%), essential minerals (0.10–916 mg/100 g dry weight), and low fat content (0.2–0.5%), with no detectable starch [6]. Additionally, previous studies revealed that date fruits contain a significant number of phytochemicals, including polyphenols, making them an ideal source of these bioactive compounds [7]. Despite the well-documented nutritional and health benefits of dates, the processing and consumption of these fruits generate substantial by-products that are often overlooked and underutilized. Innovative valorization of these by-products presents a sustainable opportunity to enhance their nutritional and functional properties, aligning with broader trends in the food industry [7,8]. The rapid expansion of the plant-based food sector has led to an increase in by-product generation, highlighting the need to address the environmental impact of waste from fruit and vegetable processing. This is essential for advancing a sustainable bioeconomy [9].

Agri-food waste, typically produced in large amounts, can be effectively exploited and transformed into high-value products, promoting more sustainable industrial practices [10]. Converting these wastes into products rich in bioactive compounds, natural chemicals, and essential nutrients not only preserves valuable resources and reduces costs but also minimizes environmental impact [11]. Achieving a sustainable bioeconomy hinges on the efficient use of resources and the production of high-quality food [12]. Consequently, innovative valorization strategies are increasingly focused on extracting valuable ingredients from fruit and vegetable by-products, allowing their use as additives or key components in functional foods, as well as in the production of bio-based nutraceutical formulations [13].

Among others, solid-state fermentation (SSF) is an innovative technique that involves microbial growth on solid substrates in the absence of free water [14]. This method has shown considerable promise in modifying the chemical profile of plant materials and plant-based products. During SSF, various carbohydrate-hydrolyzing enzymes, such as α-amylase, cellulase, β-glucosidase, and xylanase, are secreted by the microorganisms that facilitate the breakdown of covalent linkages between cell wall components and polyphenols, thereby enhancing the bioavailability of these beneficial compounds [15].

This study aims to explore the impact of solid-state fermentation using *Aspergillus niger* as a fungal strain, associated with a bacterial strain such as *Lactiplantibacillus plantarum* or *Limosilactobacillus reuteri,* on the release of phenolic compounds from by-products of three different date fruit varieties. *Aspergillus niger* was selected because of its proven efficacy in breaking down complex plant materials and increasing polyphenol content during SSF. *Lactiplantibacillus plantarum* and *Limosilactobacillus reuteri* were included in the co-culture for their probiotic properties and potential to further modify the profile of extracted bioactive compounds when combined with *Aspergillus niger*. In addition, the study examines the correlation between antioxidant and antimicrobial activities and polyphenol content in the fermented extracts. The effects of fermentation on functional groups and polyphenol profiles are also analyzed using Fourier-transform infrared spectroscopy (FT-IR) and high-performance liquid chromatography (HPLC). To the best of our knowledge, this is the first study in which both SSF and co-culture fermentation are applied to date fruit by-products, specifically aiming at enhancing polyphenol content, antioxidant power, and antimicrobial activity. This novel approach not only enhances the nutritional and functional qualities of date by-products but also offers a sustainable solution for waste management by valorizing agricultural residues. The goal of this research was to effectively exploit these by-products to develop innovative products, including nutraceuticals, pharmaceuticals, and natural additives for diverse food applications. By demonstrating the potential of date by-products, this study highlights their role as valuable resources for reducing waste and recovering bioactive compounds, contributing to the advancement of sustainable practices in the food and health sectors.

## 2. Materials and Methods

### 2.1. Plant Materials

By-products derived from three endemic date varieties, Mozafati, Sayer, and Kabkab, were obtained from local factories in Ahvaz, Iran. These by-products included non-marketable, poor-quality, and badly formed fruits. The by-products were received in batches every three months, with each batch consisting of approximately 1–2 kg of each variety. Sampling was performed in triplicate to ensure consistency and reliability. The collected samples were thoroughly washed with distilled water, cut into small pieces, and stored in a freezer until use. To ensure uniformity, by-products were selected based on comparable moisture content and physical characteristics. The samples were then lyophilized and ground using a household grinder. The resulting lyophilized powders were stored at −20 °C for up to 8 weeks prior to extraction.

### 2.2. Chemicals and Reagents

Folin–Ciocalteu phenol reagent, sodium carbonate (Na_2_CO_3_), sodium nitrate (NaNO_3_), monopotassium phosphate (KH_2_PO_4_), potassium chloride (KCl), aluminum chloride (AlCl_3_), magnesium sulfate (MgSO_4_ 2H_2_O), ferrous chloride (FeCl_2_), hydrogen peroxide (H_2_O_2_), sea salts, gallic acid, quercetin, DPPH (1,1-diphenyl-2 picrylhydrazyl), and ferrozine were obtained from Merck Chemical Co. (Darmstadt, Germany). Ethanol (96%) and methanol were provided by Hamoon Teb (Mashhad, Iran). All the other chemicals used in this study were of analytical grade.

### 2.3. Strain and Culture Medium

*Aspergillus niger* (CCM-8155) was obtained from the Iranian Biological Resource Center (Tehran, Iran). *Lactiplantibacillus plantarum* (DSM 20179) and *Limosilactobacillus reuteri* (DSM 20016) were provided by the Bioprocess Engineering Laboratory (BPEL, University of Tehran, Iran). The *Aspergillus niger* was incubated on potato dextrose agar (PDA) medium for 5 days at 30 °C. The spore suspension used for fermentation was prepared by washing the fungal colonies in the PDA plate with 0.1% Tween 80 solution.

### 2.4. Solid State Fermentation

Solid-state fermentation (SSF) was carried out in 50 mL flasks used as fermentation vessels. The culture medium composed of MgSO_4_·2H₂O (0.5 g/L), KCl (0.5 g/L), KH_2_PO_4_ (1 g/L), and NaNO_3_ (2.5 g/L), was added to the lyophilized date by-products to adjust the moisture content to 60% (*w*/*v*). For both the single and co-culture fermentations, the mixture of date by-products and culture medium was autoclaved at 121 °C for 15 min to ensure sterility. After cooling to room temperature, the substrate was inoculated with the appropriate microbial cultures as follows:

- For fungal fermentation, the substrate was inoculated with a spore suspension of *Aspergillus niger* at a concentration of 2 × 10^5^ spores/g of solid substrate.
- For the co-culture assays, *Lactiplantibacillus plantarum* and *Limosilactobacillus reuteri* were cultured overnight in MRS broth at 37 °C, then centrifuged at 6000× *g* for 10 min.

The cells were washed twice with sterile saline solution to produce washed cell suspensions, which were co-inoculated with *Aspergillus niger* into the autoclaved culture medium at a final concentration of 10^8^ CFU/mL for the bacterial strains while maintaining the spore concentration for *Aspergillus niger* as described above.

The SSF was carried out at 30 °C for 7 days, as in our previous studies it has been selected as the most suitable time to have an optimal balance between efficiency and effectiveness in terms of natural chemical content and antioxidant and antimicrobial activities [16,17]. Samples were collected at the beginning (0 h) and at the end (168 h) of the fermentation process for subsequent analysis.

### 2.5. Ultrasonic Assisted Extraction of Polyphenolic Compounds

The extraction method was performed according to Dulf et al. (2016), with some modifications [18]. Briefly, 1 g of each variety of fresh and fermented date fruit by-products was accurately weighed, and 20 mL of 70% ethanol solution was added. The samples were then placed in an ultrasonic bath (Elmasonic S 60H, Elma, Singen, Germany) and subjected to ultrasonic extraction for 30 min at 40 °C. After extraction, the samples were centrifuged at 4000× *g* for 10 min at 4 °C and then filtered. The filtrates were subjected to vacuum evaporation followed by lyophilization to remove residual moisture. The resulting powder was stored at −20 °C for further analysis, which included evaluation of total polyphenol and flavonoid content, as well as antioxidant and antimicrobial activities. All extractions were performed in triplicate to ensure accuracy and reproducibility.

### 2.6. Total Polyphenol Content

Total polyphenol content (TPC) was determined using the Folin–Ciocalteu method, as described by Alara et al. (2017) [19]. Briefly, an aliquot (100 µL) of the extract was mixed with Folin–Ciocalteu reagent (diluted 1:10) and a 7.5% (*w*/*v*) Na_2_CO_3_ solution. After incubation for 2 h at room temperature, the absorbance was measured at 760 nm against ethanol as blank. To quantify the total polyphenol content in the extract, a standard curve was prepared using gallic acid (0.05–0.2 mg/mL), and results were expressed as mg of gallic acid equivalents per g of dry weight extract (mg GA/g DW).

### 2.7. Total Flavonoid Content

Total flavonoid content (TFC) was determined using the aluminum chloride colorimetric method described by Liu et al. (2008) [20]. Briefly, 2 mL of the extract was mixed with 0.2 mL of 5% sodium nitrite and 0.2 mL of aluminum chloride. Subsequently, 2 mL of 0.1 M NaOH was added to the reaction mixture. The absorbance was measured at 510 nm against blank. Quercetin was used as the reference standard, and the total flavonoid content was expressed as mg quercetin equivalents per g of dry weight extract (mg QE/g DW).

### 2.8. DPPH Free Radical Scavenging Activity

The DPPH scavenging activity of the extracts was determined as reported by Mansouri et al., (2005) with slight modifications [21]. An aliquot of the extract (60 µL) was mixed with 1500 µL of DPPH methanolic solution (6 × 10^−5^ M). The mixture was shaken and incubated in the dark at room temperature for 30 min, after which the absorbance was measured at 515 nm. The DPPH radical scavenging activity was calculated according to Equation (1):

Scavenging activity (%) = (A_0_ − A_1_)/A_1_ × 100 (1)

where A_0_ is the absorbance of the control sample and A_1_ is the absorbance of the examined extract.

### 2.9. Metal Chelating Ability

The Fe^2+^-chelating activity was determined by measuring the formation of the Fe^2+^-ferrozine complex, as described by Decker and Welch (1990) [22]. The reaction mixture consisted of 0.5 mL of the extract (500 µg/mL), 1.6 mL of deionized water, 0.05 mL of FeCl_2_ (2 mM), and 0.1 mL of ferrozine (5 mM). In this mixture, ferrozine reacts with divalent iron to form a stable magenta complex. The mixture was incubated for 10 min at room temperature, and the absorbance was measured at 562 nm. The Fe^2+^-chelating activity of the extract was calculated according to Equation (2):

Fe^2+^ chelating ability (%) = (A_0_ − A_1_)/A_1_ × 100 (2)

where A_0_ is the absorbance of the control sample and A_1_ is the absorbance of the examined extract.

### 2.10. Hydrogen Peroxide Scavenging Activity

The hydrogen peroxide scavenging capacity of the date by-product extracts was assessed according to the method described by Ruch et al., (1989) with some minor modifications [23]. 0.6 mL of the extracts was added to 100 µL of H_2_O_2_ solution (4 mM) dissolved in phosphate-buffered saline (PBS pH 7.4) at 20 °C. After 10 min, absorbance was determined against a blank solution at 230 nm. The scavenging ability was calculated according to Equation (3):

Hydrogen peroxide scavenging activity (%) = (A_0_ − A_1_)/A_1_ × 100 (3)

where A_0_ is the absorbance of the control sample and A_1_ is the absorbance of the examined extract.

### 2.11. HPLC-PDA Analysis

The detection of the main phenolic compounds in the date by-product extracts was performed using a high-performance liquid chromatography (HPLC) system (Knauer Smartline, Knauer, Germany; 1000 pumps, PDA detector 2800, and auto sampler 3900) equipped with a C18 column (250 × 4.50 mm, particle size 5 μm; RP-ODS3, Phenomenex, Torrance, CA, USA) at room temperature. The mobile phase consisted of water containing 0.02% trifluoroacetic acid (solution A) and methanol (HPLC grade) containing 0.02% trifluoroacetic acid (solution B). The flow rate of the mobile phase was set at 0.7 mL/min. Seventeen different phenolic acids and flavonoids were analyzed, including gallic acid, 3,4-dihydroxybenzoic acid (3,4-DHB), catechin, chlorogenic acid, caffeic acid, vanillic acid, syringic acid, 2,5-dihydroxybenzoic acid (2,5-DHB), *p*-coumaric acid, ferulic acid, rutin, salicylic acid, rosmarinic acid, cinnamic acid, quercetin, kaempferol, and apigenin. These compounds were identified and quantified by comparing their retention times and spectral data with those of external standards.

### 2.12. Fourier-Transform Infrared Spectroscopy (FT-IR) Analysis

An Avatar spectrometer (Thermo Nicolet, Waltham, MA, USA) was used to evaluate the influence of fermentation on the main composition of the extract obtained from date by-products, by means of functional group characterization. For sample preparation, both fermented and unfermented date by-products were dried at 65 °C for three days and then finely ground into a powder using a grinder. The FT-IR spectra were recorded over a spectral range between 4000 and 650 cm^−1^ with a resolution of 4 cm^−1^.

### 2.13. Antimicrobial Activity

The antibacterial activities of extracts obtained from the three date by-products (Mozafati, Sayer, and Kabkab) were evaluated against three human pathogenic bacterial strains: the Gram-positive bacterium *Staphylococcus aureus* and the Gram-negative bacteria *Salmonella enterica* and *Escherichia coli*. These bacterial strains were provided by the Bioprocess Engineering Laboratory (BPEL) at the University of Tehran, Iran. Antibacterial activity was evaluated using the disk diffusion method as described by Kchaou et al. (2016) [24]. The extracts were prepared at a final concentration of 150 mg/mL by dissolving them in distilled water. Bacterial cultures were grown in nutrient broth at 37 °C overnight. For the assay, 200 µL of each bacterial culture, adjusted to approximately 10^7^ CFU/mL, was plated onto nutrient agar plates. Wells were created in the agar using a sterile borer and filled with 50 µL of the date extract solution, prepared as above. The plates were then incubated overnight at 37 °C. Gentamicin was used as a positive control for comparison. Antibacterial activity was evaluated by measuring the diameter of the inhibition halos (in millimeters).

### 2.14. Statistical Analysis

All experimental tests were performed in triplicate, and results are expressed as mean ± standard deviation (SD). A one-way ANOVA followed by Tukey’s honestly significant difference (HSD) test for multiple comparisons was used to compare the means between different groups. Statistical analyses were performed using the R version 4.4.0 software package (www.r-project.org (accessed on 1 September 2024)). Pearson correlation analysis was also used to evaluate the relationships between the concentrations of bioactive compounds and antioxidant activities.

## 3. Results and Discussion

### 3.1. Total Phenolic and Flavonoid Content

The mean values of total polyphenol content (TPC) and total flavonoid content (TFC) for all extracts ranged from 3.07 ± 0.07 to 12.98 ± 0.29 mg GA/g DW and from 0.18 ± 0.04 to 1.83 ± 0.07 mg QE/g DW, respectively (Table 1). Among the unfermented samples, Sayer had the highest TPC and TFC (4.64 ± 0.07 mg GA/g DW and 0.33 ± 0.06 mg QE/g DW), followed by Mozafati (3.69 ± 0.07 mg GA/g DW and 0.26 ± 0.04 mg QE/g DW) and Kabkab (3.07 ± 0.07 mg GA/g DW and 0.18 ± 0.04 mg QE/g DW), respectively.

These findings are consistent with those verified by Saafi et al. (2009), who reported that the TPC of date varieties from Tunisia ranged from 209 to 448 mg GA/100 g DW extract [25]. Benmeddour et al. (2013) also measured the TPC and TFC of ten unfermented Algerian date cultivars, with values ranging from 225.57 ± 9.71 to 947.56 ± 25.32 mg GA/100 g DW extract and 15.22 ± 0.50 to 299.74 ± 5.87 mg QE/100 g DW extract, respectively [6]. However, it is key to acknowledge that all the above-mentioned studies were focused on the analyses of the content of mature dates with uniform size, free of physical damage, insect injury, and fungal infection, which may be richer in active molecules in comparison with date by-products that are non-marketable and of poor quality tested in this study.

Fermentation using filamentous fungi has been demonstrated to significantly improve the phenolic content and antioxidant activity of plant-based matrices and fermented foods, depending on the microorganisms involved [26,27]. As a confirmation, fermentation with *Aspergillus niger* significantly increased the TPC and TFC of tested date by-products. In addition, among the fermented samples, co-culture fermentations with *Lactiplantibacillus plantarum* and *Limosilactobacillus reuteri* exhibited the highest content of polyphenols, whereas single cultures of all three varieties had the lowest. The maximum increase in the TPC and TFC was achieved in the fermented sample of Mozafati by-product using co-culture fermentation of *Aspergillus niger* with *Lactiplantibacillus plantarum* (12.98 ± 0.29 mg GA/g DW extract), while the highest TFC was achieved using the Sayer variety (1.83 ± 0.07 mg QE/g DW extract), using the same co-culture fermentation. The effect of fermentation with different microorganisms on the polyphenol content of various plant substrates and agricultural wastes has been discussed by several authors. Xiao et al. (2014) reported that the total polyphenol and total saponin contents of fermented chickpeas with *Cordyceps militaris* were considerably higher than those of unfermented ones [28]. Similarly, Yeo et al. (2021) observed an increase in the insoluble polyphenol content in lentil hulls fermented with *Rhizopus oryzae* [29]. Additionally, co-culture fermentation is recognized for its enhanced ability to release bound polyphenols from cell wall components more effectively. For instance, Khan et al. (2020) found that co-culture fermentation of extruded rice bran with *Lactiplantibacillus plantarum*, *Lactiplantibacillus fermentum*, and *Saccharomyces cerevisiae* significantly increased TPC from 122.83 ± 1.15 to 237.46 ± 7.74 mg GA/100 g DW extract and TFC from 67.86 ± 3.75 to 109.68 ± 6.02 mg CE/100 g DW extract. During SSF, microorganisms such as filamentous fungi and bacteria secrete various enzymes that modify polyphenol profiles. Specifically, esterase and xylanase enzymes hydrolyze covalent bonds between polyphenols and cell wall components, while protease and cellulase break down structural cell wall components of date by-products thus improving the bioavailability of polyphenols [30].

### 3.2. Antioxidant Activities

Antioxidants are essential for protecting macromolecules from oxidation damage that could impair their function. Therefore, the evaluation of antioxidant activity is crucial for assessing the quality of date fermentation [31]. The antioxidant capacity of date by-product extracts was measured using multiple in vitro assays, including radical scavenging activity (DPPH and hydrogen peroxide scavenging) and metal chelating ability.

#### 3.2.1. DPPH

As reported in Table 2, fermentation significantly (*p* < 0.05) increased the DPPH radical scavenging ability of the extracts, reaching the highest level of 84.4 ± 1.18% in the co-culture fermentation of *Aspergillus niger* and *Lactiplantibacillus plantarum* of the Mozafati variety, probably due to its higher phenolic content. The Sayer variety, fermented using co-culture with *Lactiplantibacillus plantarum* or with *Limosilactobacillus reuteri,* achieved 80.4 ± 1.37% and 79.23 ± 1.82%, respectively. These results suggest that the fermentation process, rather than the specific date by-product, primarily contributed to the enhancement of scavenger activity of the extract.

Results suggest that co-culture systems can significantly increase the antioxidant activity of extracts. This increase is associated with higher phenolic content, primarily due to the enhanced ability of the process to convert bound and conjugated polyphenols into free forms, compared to single cultures [32]. This in turn leads to an improvement of hydroxyl groups commonly present in these molecules, which are considered to be responsible for this beneficial activity. The observed DPPH scavenging activity of the date by-products aligns with the findings mentioned by Luo et al. (2020), who concluded that the mixed-culture fermentation of corn seed with *M. anka*, *S. cerevisiae*, and *B. subtilis* can significantly enhance the DPPH and ABTS scavenging activity of fermented samples [33].

#### 3.2.2. Metal Chelating Activity

As reported in Table 2, the extracts of both fermented and unfermented samples exhibited significant (*p* < 0.05) differences in their Fe^2+^ chelating capacity, depending on both the type of date and the microorganisms used. The chelating capacity ranged from 23.36 ± 0.94% to 74.33 ± 1.21%. Furthermore, the results demonstrated that the fermented samples had a stronger ability to chelate Fe^2+^ compared to the unfermented ones. The highest chelating activity (74.33 ± 1.21%) was achieved for the Sayer variety co-fermented with *Aspergillus niger* and *Lactiplantibacillus plantarum*. These results align with those of Sadh et al. (2018), who reported a threefold increase in chelating activity of peanut press cake following fermentation [34]. In addition, other researchers have found that solid-state fermentation has a prominent impact on the ability of samples to chelate Fe^2+^, although the extent of this effect varies depending on the species of microorganism and the type of by-product [35,36].

#### 3.2.3. H_2_O_2_ Scavenging Ability

The H_2_O_2_ scavenging ability of all date by-product extracts ranged from 37.26 ± 0.57% to 51.96 ± 0.60%, with the fermented extract of the Sayer by-product with *Aspergillus niger* and *Limosilactobacillus reuteri* exhibiting the highest value (Table 2). Following fermentation, the date by-product extracts showed enhanced effectiveness in scavenging hydrogen peroxide, consistent with findings reported by other authors. Abbes et al. (2013) observed an increased hydrogen peroxide scavenging activity of extracts obtained from three Tunisian date varieties through the enzymatic treatment using a mixture of cellulase and pectinase [37]. In this case, fermentation does not seem to exert an effective improvement in hydrogen peroxide scavenging activity compared to the unfermented samples, as observed for DPPH scavenging and metal chelating abilities. Furthermore, it was revealed that the hydrogen peroxide scavenging ability of the fermented extracts did not correlate with the improvement in TPC and TFC. For instance, the Mozafati extract co-cultured with *Aspergillus niger* and *Limosilactobacillus reuteri* exhibited the lowest level of H_2_O_2_ scavenging activity despite its high polyphenol and flavonoid content. These findings suggest that the samples may be strongly affected by the presence of various antioxidant compounds, including ascorbic acid, tocopherol, and pigments. It is possible that the fermented date by-product extracts contain compounds with relatively weaker hydrogen peroxide scavenging abilities [2].

### 3.3. Correlations

**Figure 1:**
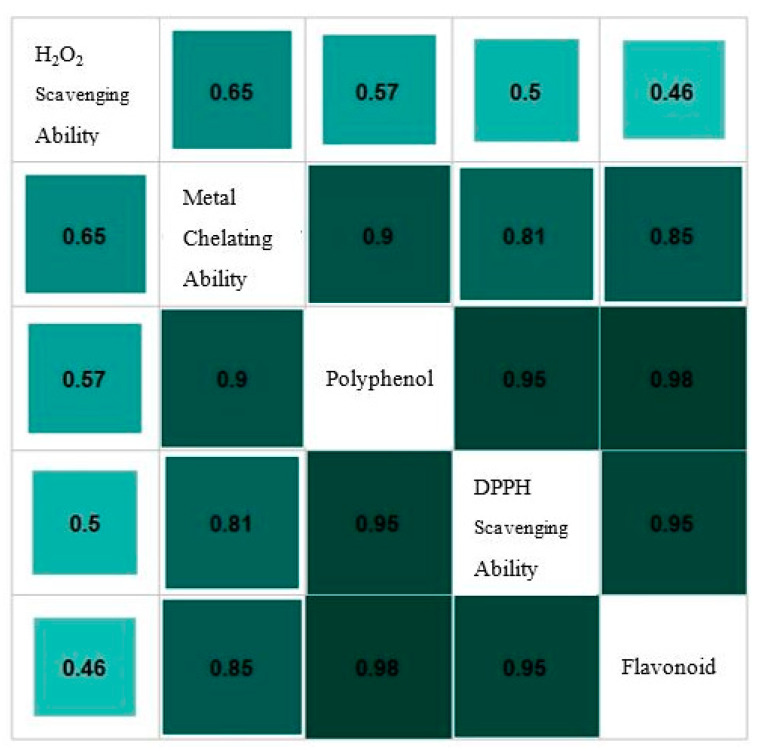
Correlation between total polyphenol, flavonoid, and antioxidant activities, including DPPH radical scavenging ability, H_2_O_2_ scavenging activity, and metal chelating ability. Darker colors indicate stronger correlations between variables.

The Pearson correlation test was employed to analyze the correlation between the phenolic and flavonoid contents and antioxidant activities of different date by-product extracts. TPC and TFC exhibited a strong correlation with DPPH radical scavenging activity (r = 0.951 and r = 0.982, respectively) and metal chelating ability (r = 0.899 and r = 0.854, respectively). However, there was only a weak correlation (r = 0.566 and r = 0.459, respectively) between H_2_O_2_ scavenging ability and the TPC and TFC of the extracts, suggesting that these bioactive compounds might not be the primary contributors to the extracts’ ability to scavenge H_2_O_2_. Figure 1 shows information on the distribution of antioxidant properties and polyphenol concentration in the date by-products. Darker colors indicate stronger correlations between variables. These results are consistent with Benmeddour et al. (2013) who determined the correlation between total polyphenols, total flavonoids, total flavonol, and total condensed tannin with the antioxidant activities of Algerian date fruits [6]. Similarly, Carmo Brito et al. (2017) revealed a strong positive correlation between total phenolic compounds and total anthocyanins with the ABTS scavenging activity of fermented cocoa beans [38].

### 3.4. Profile of Polyphenols

HPLC coupled with a UV detector has been used to assess the profile and concentration of the main polyphenols contained in the extracts before and after both single and co-culture fermentations (Table 3). Seven main phenolic compounds were identified in date by-product varieties, including ferulic acid, *p*-coumaric acid, caffeic acid, gallic acid, kaempferol, rutin, and quercetin.

In all unfermented extract obtained from date by-products, ferulic acid was the most abundant compound (0.830 ± 0.013 to 0.973 ± 0.030 mg/g of extract), followed by p-coumaric acid (0.583 ± 0.023 to 0.813 ± 0.021 mg/g of extract), caffeic acid (0.580 ± 0.010 to 0.717± 0.020 mg/g of extract), gallic acid (0.367 ± 0.013 to 0.607 ± 0.007 mg/g of extract), kaempferol (0.123 ± 0.009 to 0.200 ± 0.018 mg/g of extract), and quercetin (0.0467 ± 0.006 to 0.113 ± 0.012 mg/g of extract), with the highest amounts found in the Sayer variety. In agreement with these findings, Al-Farsi et al. (2005) found that ferulic acid was the main phenolic acid in the Omani date variety. They also demonstrated that the phenolic acid content was higher than that of flavonoids in the examined date extracts [7]. A similar phenolic acid profile was observed by Harthi et al. (2015), who showed that the main phenolic acids found in four Omani date varieties were gallic acid, caffeic acid, *p*-coumaric acid, and vanilic acid [39]. In contrast, a study conducted by Bouhlali et al. (2018) showed that gallic acid was the predominant polyphenol found in Moroccan date fruit cultivars; however, the profile of phenolic compounds was nearly the same [40]. Although the content of various polyphenols differed significantly (*p* < 0.05) among unfermented samples, all these three varieties displayed a nearly identical phenolic profile with minor variations. This consistency may be attributed to their cultivation under similar environmental conditions, including consistent soil and water quality, within the same region, although they were not grown in the same crop [41]. As shown in Table 3, the profile of individual polyphenols was significantly different after single and co-culture fermentations. Several studies have attributed these alterations to the metabolic activity of microorganisms [42]. In the single cultures of all three varieties fermented with *Aspergillus niger*, *p*-coumaric acid was the prevalent polyphenol with concentrations ranging from 3.410 ± 0.080 to 4.490 ± 0.940 mg/g. Despite the significant increases in caffeic acid (2.040 ± 0.046 to 2.690 ± 0.028 mg/g), kaempferol (0.417 ± 0.015 to 0.507 ± 0.051 mg/g), and quercetin (0.260 ± 0.055 to 0.443 ± 0.006 mg/g) levels, after fermentation in the single cultures (*p* < 0.05), there were notable decreases in gallic acid and ferulic acid. A significant change observed in co-culture systems was the complete disappearance of gallic acid in all three varieties. This may be due to the activity of tannase and gallate decarboxylase enzymes present in both *Lactiplantibacillus* and *Limosilactobacillus* species and *Aspergillus niger*, which convert gallic acid into pyrogallol and other derivatives [43,44,45]. Jiménez et al., (2014) discussed the metabolic pathways involving gallate decarboxylase and tannase enzymes in *Lactiplantibacillus plantarum* species that are responsible for the degradation of gallic acid [46]. Arentshorst et al. (2021) investigated the expression of genes regulating tannase in *Aspergillus niger*, which are crucial for the catabolism of gallic acid into gallic acid lactone (GAL) in the fermentation medium. This metabolism is facilitated by specific ring-cleaving enzymes that break the ring structure of gallic acid, converting it into compounds such as pyrogallol or methyl gallate [47]. The concentrations of ferulic acid also decreased after the fermentation process across all varieties. This reduction can be attributed to the enzymatic activities of the microorganisms involved in the fermentation [28]. Hegde et al. (2006) reported that the content of ferulic acid decreased in wheat bran after fermentation with *Aspergillus niger* due to the activity of ferulic acid esterase that can convert this phenolic acid to *p*-coumaric acid [48]. The reduction in gallic acid and ferulic acid content in fermented substrates can be explained by the microbial degradation, reduction, or oxidation of these compounds by the fermenting microorganisms. Furthermore, enzymatic bioconversion occurring during solid-state fermentation may also lead to a decrease in certain phenolic acid levels. Such alterations in phenolic acid profiles are highly influenced by various factors, including the substrate type, fungal species employed, and the specific conditions of fermentation [49]. In both co-culture fermentation processes, rutin, was detected by HPLC as the main flavonoid compound, with concentrations ranging from 2.110 ± 0.097 to 3.310 ± 0.092 mg/g. The observed increase in rutin content may be due to the action of microbial enzymes, such as glycosidases and esterases, which release rutin from complex plant matrices during the fermentation process [2]. Similarly, Xiao et al., 2014 reported that the content of individual polyphenols, including rutin, was increased after fermentation [28]. Randhir and Shetty (2007) highlighted the significant role of β-glucosidase in mobilizing phenolic compounds from mung bean substrates during fungal bioprocessing with *Rhizopus oligosporus*. They noted a direct correlation between increased β-glucosidase activity and the release of phenolics [50]. In addition, employing co-culture systems enhances the synergistic activities among microorganisms, resulting in more significant changes in the content and profile of individual polyphenols [51]. These modifications in the polyphenol profile are significant because they not only enhance the antioxidant properties of the date by-products but also expand their potential applications in the food and nutraceutical industries. For instance, the enriched polyphenolic content, particularly rutin, could be leveraged as a natural additive in food products to improve shelf life and nutritional value, or in nutraceuticals as a functional ingredient with health-promoting properties. Furthermore, the reduction in ferulic and gallic acid through fermentation may lead to products with tailored antioxidant profiles, which could be beneficial in specific food formulations or health supplements [40].

### 3.5. FT-IR Analysis

**Figure 2:**
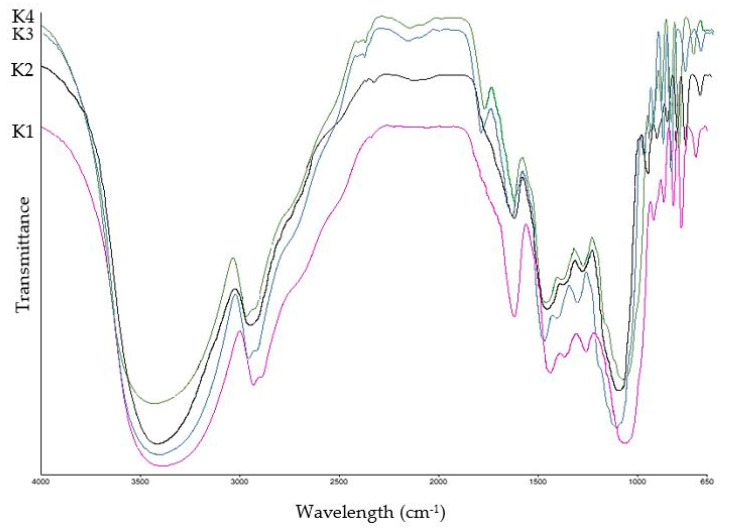
FT-IR spectra of Kabkab date by-product samples. K1 unfermented sample; K2 single culture using *Aspergillus niger*; K3 co-cultured samples using *Aspergillus niger* and *Limosilactobacillus reuteri*; K4 co-cultured samples using *Aspergillus niger* and *Lactiplantibacillus plantarum*.

**Figure 3:**
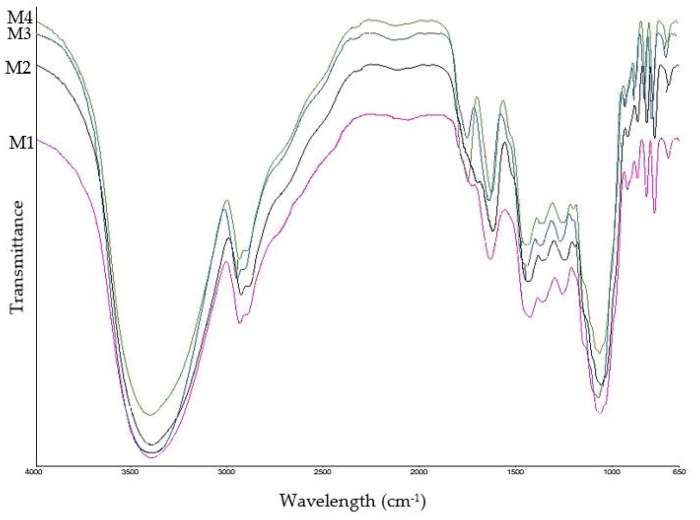
FT-IR spectra of Mozafati date by-product samples. M1: unfermented sample; M2: single culture using *Aspergillus niger*; M3: co-cultured samples using *Aspergillus niger* and *Limosilactobacillus reuteri*; M4: co-cultured samples using *Aspergillus niger* and *Lactiplantibacillus plantarum*.

**Figure 4:**
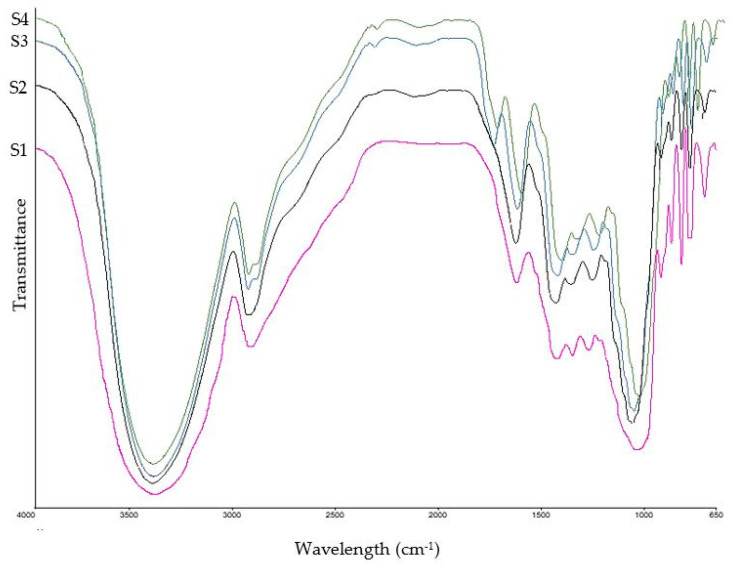
FT-IR spectra of Kabkab date by-product samples. S1: unfermented sample; S2: single culture using *Aspergillus niger*; S3: co-cultured samples using *Aspergillus niger* and *Limosilactobacillus reuteri*; S4: co-cultured samples using *Aspergillus niger* and *Lactiplantibacillus plantarum*.

Figure 2, Figure 3 and Figure 4 and Table 4 detail the changes in chemical bonds and functional groups observed in all date by-product samples. Additionally, Table 5 lists the various functional groups and their corresponding organic compounds identified across all tested samples.

As can be seen in Table 5, dates are mainly composed of simple carbohydrates, including glucose, fructose, and sucrose, as well as fibers such as cellulose, hemicellulose, lignin, and pectin. Additionally, they contain minerals, carotenoids, polyphenols (both phenolic acids and flavonoids), vitamins, and proteins [52]. Strong absorption bands were detected around 3390 cm^−1^, indicating O–H stretching vibrations within the polysaccharide sugar chain and polyphenolic compounds. The peaks observed at 2930–2934 cm^−1^ and 1421–1442 cm^−1^ are assigned to methylene and methyl groups in polysaccharides and proteins. The peaks at 1631–1635 cm^−1^ represent aromatic rings in lignin, C=C bonds in polyphenols, and carotenoids in date by-products. The bands at 1245–1257 cm^−1^ correspond to C–O stretching in hemicellulose, polysaccharides, and phenolic compounds. The peaks at 1047–1056 cm^−1^ describe glycosidic bonds in cellulose and hemicellulose, as well as carboxylic acids. Signals at wavelengths under 1000 cm^−1^ represent structural components, including lignin, cellulose, and hemicellulose [53,54]. The FT-IR spectra revealed notable changes in the functional groups of compounds in all date varieties during the fermentation process [55]. Increased absorption intensities were observed, along with the appearance of new peaks and slight shifts in peak positions after fermentation. In all date by-product varieties, the intense absorption ranging from 1737 to 1745 cm^−1^ was observed after the co-culture fermentation process, which corresponds to the carbonyl group stretching vibration characteristic of ester groups found in ketones derived from flavonoids and phenolic acids. This can elucidate the significant enhancement in the content of these bioactive compounds after fermentation [56]. These changes in the FT-IR spectra of fermented samples are mainly attributed to the mentioned substantial biochemical changes within the date by-products, including an enhancement in polyphenol concentrations, alterations in polysaccharide structures, and the formation of new organic compounds [57].

**Table 5:** Assignment of principal IR absorption bands in the date by-products of three varieties before and after fermentation.

Observed Wavelength (cm^−1^)	Assignment	Description	Specific Organic Compounds	Reference
3370–3399	O-H stretching	sugars, polyphenols	Hydroxyl groups in polyphenols, sugars, and water	[57]
2930–2934	C-H stretching	Alkanes	Methylene and methyl groups in polysaccharides and proteins	[55]
1737–1745	C=O stretching	Carbonyl groups (esters, ketones, aldehydes)	Ester groups in hemicellulose, ketones from degradation products such as flavonoids and phenolic acids	[58]
[55,56]	C=C stretching	Alkenes, aromatic rings	Aromatic rings in lignin, C=C in polyphenols, carotenoids	[56,57]
1421–1442	C-H bending	Alkanes	Methylene and methyl groups in polysaccharides	[55]
1245–1257	C-O stretching in C–C(=O)–O bonds	Esters, ethers, polyphenols	Ester groups in hemicellulose, ethers in polysaccharides, phenolic ethers	[59]
1047–1056	C-O stretching in O–C–C bonds	Alcohols, ethers, carboxylic acids	Glycosidic bonds in cellulose and hemicellulose, primary and secondary alcohols	[58]
917–919	C-H rocking/bending vibration	Carbohydrates	Cellulose and hemicellulose	[54]
866–867	C-H bending/rocking vibration	Carbohydrates	Cellulose and hemicellulose	[54]
818–819	C-H bending/rocking vibration	Aromatics	Aromatic rings in lignin	[52]
778–779	C-H rocking vibration	Carbohydrates	Cellulose and hemicellulose	[52]
705–706	C-H rocking vibration	Carbohydrates	Cellulose and hemicellulose	[52]

### 3.6. Antimicrobial Activity

Extracts from three selected date varieties were tested for antimicrobial activity against two Gram-positive strains (*Staphylococcus aureus and Salmonella enteritidis*) and a Gram-negative strain (*Escherichia coli*). The diameters of inhibition halos are given in Table 6. The findings revealed that both unfermented and fermented extracts inhibited the growth of microorganisms in all tested samples. According to Table 6, co-fermented samples showed a slightly higher antibacterial activity against pathogens, particularly Gram-positive bacteria. Furthermore, co-cultured samples demonstrated slightly superior efficacy against pathogenic bacteria compared to single culture. Among the three varieties, the extract from co-culture fermentation with *Aspergillus niger* and *Lactiplantibacillus plantarum* provided the largest inhibition halo against *Staphylococcus aureus*, with values ranging from 10.2 ± 0.08 to 17.73 ± 0.09 mm. Unfermented extracts from the Kabkab variety had no inhibitory impact on any of the three tested microorganisms. On the contrary, the Kabkab variety co-fermented with *Aspergillus niger* and *Lactiplantibacillus palntarum* exhibited the largest inhibitory zone (16.26 ± 0.15 mm) against *Salmonella enteritidis*. Additionally, for co-fermented date by-products derived from the Mozafati and Sayer varieties, the largest inhibition zones were achieved against *Staphylococcus aureus* and *Escherichia coli* with diameters of 17.73 ± 0.09 mm and 15.7 ± 0.09 mm, respectively. These findings are in line with those reported by Daoud et al. (2019), who investigated the antimicrobial effects of date palm pollen extracts against different Gram (+) and Gram (−) bacteria, including *Bacillus cereus*, *Staphylococcus aureus*, *Listeria monocytogenes*, *Salmonella Enteritidis*, *Salmonella typhimurium*, and *Escherichia coli*, whose highest inhibitory effect was found against *Staphylococcus aureus* (15.5 ± 0.5 mm) [60]. The variability observed in the efficacy of unfermented, fermented, and co-fermented extracts against the microorganisms tested can be attributed to the differences in their composition, mainly due to the cultivar tested and the process used to extract the active compounds. In fact, several factors, such as plant extraction solvents, plant parts used, extraction methods, environmental microorganisms, plant growing regions, and plant harvesting seasons, strongly influence the antimicrobial activity of different fruits [61]. In addition, Gram-positive and Gram-negative bacteria may have different sensitivities to the antimicrobial activity of the extracts due to differences in cell membrane components and cell wall structure [62].

## 4. Conclusions

In this study, the polyphenol content, antioxidant activity, and antimicrobial properties of extracts from date by-products of three different varieties, as well as the effect of co-culture fermentation with *Aspergillus niger* and lactic acid bacteria, were investigated. Co-culture fermentation significantly improved the polyphenol and flavonoid content, resulting in a marked increase in antioxidant activity. In particular, the most significant improvements were observed with the co-fermentation of *Aspergillus niger* and *Lactiplantibacillus plantarum*, as evidenced by high-performance liquid chromatography (HPLC) and Fourier transform infrared spectroscopy (FT-IR) analyses, which revealed significant changes in the functional groups and polyphenolic profiles of the fermented extracts. The increased levels of bioactive compounds, such as rutin, highlight the potential of these co-fermented extracts as valuable additives in the nutraceutical, pharmaceutical, and food industries. In addition, the antimicrobial efficacy of the extracts against both Gram-positive and Gram-negative bacteria increased significantly, with the most pronounced effects observed after co-fermentation. Of note, *Aspergillus niger* and *Lactiplantibacillus plantarum* are recognized as Generally Regarded as Safe (GRAS) and thus ideal for the development of additives to be included in functional foods with extended shelf life or in nutraceuticals aimed at providing health benefits thanks to their antioxidant power.

To our knowledge, this is the first study to explore the effects of co-culture fermentation on the beneficial properties of Iranian date by-products. These findings provide new insights into how co-culture fermentation can enhance the biological functions of polyphenols in date by-products, highlighting their potential for increased value and functionality in the food industry and beyond. Furthermore, this research underscores the broader implications for environmental sustainability and human health, positioning these upgraded co-products as key components in the development of a sustainable bioeconomy.

## Figures and Tables

**Table 1:** Total polyphenol content (TPC) and Total flavonoid content (TFC) of unfermented and fermented date by-product extracts. Data are expressed as mean ± SD. For each group, means with different lowercase letters show a significant difference (*p* < 0.05).

Sample	TPC (mg GA/g DW)	TFC (mg QE/g DW)
Kabkab		
K1	3.07 ± 0.07 ^j^	0.18 ± 0.04 ^g^
K2	7.78 ± 0.28 ^g^	0.81 ± 0.10 ^f^
K3	8.93 ± 0.11 ^f^	1.12 ± 0.09 ^e^
K4	10.84 ± 0.25 ^d^	1.34 ± 0.05 ^d^
Mozafati		
M1	3.69 ± 0.07 ^i^	0.26 ± 0.04 ^g^
M2	9.55 ± 0.16 ^e^	1.09 ± 0.09 ^e^
M3	11.15 ± 0.19 ^cd^	1.57 ± 0.05 ^bc^
M4	12.98 ± 0.29 ^a^	1.75 ± 0.07 ^ab^
Sayer		
S1	4.64 ± 0.07 ^h^	0.33 ± 0.06 ^g^
S2	9.21 ± 0.30 ^ef^	0.93 ± 0.08 ^ef^
S3	11.56 ± 0.10 ^c^	1.54 ± 0.05 ^cd^
S4	12.16 ± 0.04 ^b^	1.83 ± 0.07 ^a^

K1, M1, and S1: unfermented samples; K2, M2, and S2: single culture using *Aspergillus niger*; K3, M3, and S3: co-cultured samples using *Aspergillus niger* and *Limosilactobacillus reuteri*; K4, M4, and S4: co-cultured samples using *Aspergillus niger* and *Lactiplantibacillus plantarum*.

**Table 2:** DPPH radical scavenging activity, metal chelating ability, and H_2_O_2_ scavenging ability of various date by-product extracts. Data are expressed as mean ± SD. For each group, means with different lowercase letters show a significant difference (*p* < 0.05).

Sample	DPPH Scavenging Activity (%)	Metal Chelating Ability (%)	H_2_O_2_ Scavenging Ability (%)
Kabkab			
K1	28.23 ± 0.97 ^g^	23.36 ± 0.94 ^g^	37.26 ± 0.57 ^g^
K2	41.30 ± 1.25 ^f^	54.53 ± 1.40 ^de^	48.60 ± 0.10 ^c^
K3	55.73 ± 1.40 ^e^	67.60 ± 1.22 ^c^	47.86 ± 0.45 ^c^
K4	70.43 ± 1.22 ^c^	69.70 ± 0.55 ^bc^	42.46 ± 0.65 ^de^
Mozafati			
M1	40.13 ± 0.87 ^f^	25.63 ± 1.18 ^fg^	40.43 ± 0.80 ^ef^
M2	60.40 ± 1.77 ^d^	52.10 ± 1.49 ^e^	48.40 ± 0.65 ^c^
M3	69.50 ± 1.05 ^c^	55.26 ± 0.72 ^de^	38.73 ± 0.85 ^fg^
M4	84.40 ± 1.18 ^a^	72.10 ± 1.28 ^ab^	49.40 ± 0.89 ^bc^
Sayer			
S1	43.26 ± 1.20 ^f^	28.40 ± 1.51 ^f^	38.46 ± 1.29 ^fg^
S2	62.43 ± 0.92 ^d^	70.53 ± 1.30 ^bc^	51.53 ± 0.87 ^ab^
S3	80.40 ± 1.37 ^b^	57.66 ± 0.78 ^d^	51.96 ± 0.60 ^a^
S4	79.23 ± 1.82 ^b^	74.33 ± 1.21 ^a^	44.13 ± 0.87 ^d^

K1, M1, and S1: unfermented samples; K2, M2, and S2: single culture using *Aspergillus niger*; K3, M3, and S3: co-cultured samples using *Aspergillus niger* and *Limosilactobacillus reuteri*; K4, M4, and S4: co-cultured samples using *Aspergillus niger* and *Lactiplantibacillus plantarum*.

**Table 3:** Phenolic composition of date by-product extracts before and after fermentation. Data are expressed as mean ± SD. For each group, means with different lowercase letters in show a significant difference (*p* < 0.05).

Sample	Gallic Acid (mg/g)	Caffeic Acid (mg/g)	*p*-Coumaric Acid (mg/g)	Ferulic Acid (mg/g)	Rutin (mg/g)	Quercetin (mg/g)	Kampferol (mg/g)
Kabkab							
K1	0.367 ± 0.013 ^e^	0.580 ± 0.010 ^i^	0.583 ± 0.023 ^i^	0.830 ± 0.013 ^c^	-	0.047 ± 0.006 ^j^	0.123 ± 0.031 ^i^
K2	0.140 ± 0.005 ^f^	2.040 ± 0.046 ^g^	3.410 ± 0.080 ^g^	0.317 ±0.043 ^e^	-	0.260 ± 0.055 ^g^	0.430 ± 0.018 ^g^
K3	-	2.330 ± 0.150 ^f^	4.210 ± 0.068 ^f^	0.123 ± 0.025 ^hi^	2.110 ± 0.097 ^e^	0.427 ± 0.028 ^e^	0.583 ± 0.039 ^e^
K4	-	2.620 ± 0.085 ^d^	4.340 ± 0.120 ^e^	0.153 ± 0.010 ^gh^	2.720 ± 0.190 ^c^	0.460 ± 0.017 ^de^	0.593 ± 0.048 ^e^
Mozafati							
M1	0.577 ± 0.015 ^b^	0.647 ± 0.050 ^h^	0.770 ± 0.010 ^h^	0.890 ± 0.020 ^b^	-	0.070 ± 0.001 ^i^	0.123 ± 0.009 ^h^
M2	0.420 ± 0.010 ^d^	2.510 ± 0.080 ^e^	4.290 ± 0.150 ^e^	0.253 ± 0.041 ^f^	-	0.443 ± 0.006 ^e^	0.507 ± 0.051 ^f^
M3	-	2.610 ± 0.053 ^d^	4.440 ± 0.083 ^d^	0.163 ± 0.074 ^g^	2.630 ± 0.220 ^d^	0.490 ± 0.024 ^cd^	0.653 ± 0.072 ^d^
M4	-	2.810 ± 0.180 ^b^	4.870 ± 0.074 ^a^	0.123 ± 0.052 ^hi^	3.310 ± 0.092 ^a^	0.637 ± 0.011 ^a^	0.780 ± 0.035 ^b^
Sayer							
S1	0.607 ± 0.007 ^a^	0.717± 0.020 ^a^	0.813 ± 0.021 ^h^	0.973 ± 0.030 ^a^	-	0.113 ± 0.012 ^h^	0.200 ± 0.018 ^h^
S2	0.490 ± 0.015 ^c^	2.690 ± 0.028 ^c^	4.490 ± 0.940 ^cd^	0.393 ± 0.024 ^d^	-	0.333 ± 0.009 ^f^	0.417 ± 0.015 ^g^
S3	-	2.760 ± 0.070 ^b^	4.540 ± 0.055 ^c^	0.107 ± 0.018 ^i^	2.710 ± 0.036 ^c^	0.523 ± 0.021 ^c^	0.710 ± 0.027 ^c^
S4	-	2.940 ± 0.084 ^a^	4.650 ± 0.080 ^b^	0.103 ± 0.009 ^i^	2.800 ± 0.210 ^b^	0.567 ± 0.033 ^b^	0.833 ± 0.005 ^a^

K1, M1, and S1: unfermented samples; K2, M2, and S2: single culture using *Aspergillus niger*; K3, M3, and S3: co-cultured samples using *Aspergillus niger* and *Limosilactobacillus reuteri;* K4, M4, and S4: co-cultured samples using *Aspergillus niger* and *Lactiplantibacillus plantarum.*

**Table 4:** The vibrational frequencies of functional groups detected in the FT-IR spectra of three different date by-product varieties analyzed in the spectral range of 3400–700 cm^−1^.

Sample	OH Stretching (3600–3200 cm^−1^)	C-H Stretching (3000–2800 cm^−1^)	C=O Stretching (1750–1680 cm^−1^)	C=C Stretching (1680–1620 cm^−1^)	C-H Stretching (1600–1400 cm^−1^)	C-O Stretching (1300–1000 cm^−1^)	C-H Stretching (920–705 cm^−1^)
M1	3391.76	2932.74	-	1631.18	1425.25	1256.9, 1056.69	917.74, 866.53, 778.05, 705.91
M2	3393.23	2927.54	-	1629.62	1432.47	1254.3, 1056.06	918.66, 866.43, 818.06, 778.07, 705.83
M3	3399.1	2931.98	1740.92	1622.3	1429.84	1245.64, 1047.54	918.23, 867.91, 818.11, 778.02, 705.32
M4	3398.61	2934.22	1744.02	1619.28	1433.94	1245.12, 1049.19	918.32, 867.16, 818.26, 778.1, 705.07
K1	3394.32	2931.11	-	1629.48	1421.72	1257.14, 1055.61	918.27, 866.22, 818.2, 777.83, 705.06
K2	3394.72	2931.32	-	1628.32	1439.42	1256.73, 1055.61	918.24, 866.69, 818.16, 777.75, 705.11
K3	3395.31	2932.17	1743.05	1627.54	1442.14	1250.62, 1055.97	919.03, 867.22, 818.19, 778.86, 705.37
K4	3396.58	2932.68	1738.12	1624.77	1439.63	1253.07, 1056.32	919.41, 867.23, 818.16, 778.19, 705.42
S1	3370.72	2930.24	-	1634.9	1427.8	1256.34, 1056.18	917.84, 866.39, 818.57, 777.79, 705.93
S2	3383.78	2931.52	-	1629.43	1428.04	1255.93, 1056.22	918.04, 866.51, 818.55, 777.83, 705.65
S3	3389.34	2931.62	1742.13	1624.33	1428.03	1254.69, 1056.27	918.23, 866.58, 818.43, 777.83, 705.48
S4	3393.17	2933.32	1737.95	1623.92	1428.14	1255.04, 1056.53	918.42, 866.76, 818.2, 777.91, 705.52

K1, M1, S1: unfermented samples; K2, M2, S2: single culture fermentation using *Aspergillus niger*; K3, M3, S3: co-culture fermentation using *Aspergillus niger* and *Limosilactobacillus reuteri*; K4, M4, S4: co-culture fermentation using *Aspergillus niger* and *Lactiplantibacillus plantarum* derived from date by-product samples of the Kabkab (K), Mozafati (M), and Sayer (S) varieties, respectively.

**Table 6:** Antimicrobial activity of date by-product extracts against Gram-negative and Gram-positive bacterial strains. Data are expressed as means ± SD. For the same group, means with different lowercase letters indicate significant differences between the different treatments at a significance level of (*p* < 0.05). nd: not determined.

	*Salmonella enteritidis*	*Escherichia coli*	*Staphylococcus aureus*
	Inhibition Zone (mm)	Inhibition Zone (mm)	Inhibition Zone (mm)
Kabkab			
K1	nd	nd	nd
K2	8.53 ± 0.14 ^h^	8.76 ± 0.11 ^h^	8.26 ± 0.06 ^i^
K3	9.30 ± 0.11 ^g^	9.20 ± 0.10 ^g^	9.66 ± 0.16 ^f^
K4	10.56 ± 0.15 ^f^	10.60 ± 0.12 ^f^	10.20 ± 0.08 ^g^
Mozafati			
M1	8.20 ± 0.12 ^i^	8.36 ± 0.11 ^i^	8.24 ± 0.20 ^i^
M2	11.86 ± 0.20 ^d^	10.33 ± 0.11 ^f^	12.30 ± 0.05 ^e^
M3	15.43 ± 0.21 ^c^	15.90 ± 0.10 ^c^	17.20 ± 0.16 ^c^
M4	16.26 ± 0.15 ^b^	16.70 ± 0.14 ^b^	17.73 ± 0.09 ^b^
Sayer			
S1	8.23 ± 0.05 ^i^	8.24 ± 0.09 ^i^	8.33 ± 0.11 ^i^
S2	11.26 ± 0.15 ^e^	11.40 ± 0.11 ^e^	11.80 ± 0.11 ^f^
S3	15.10 ± 0.17 ^c^	14.20 ± 0.15 ^d^	15.27 ± 0.05 ^d^
S4	15.26 ± 0.15 ^c^	15.70 ± 0.09 ^c^	15.56 ± 0.12 ^d^
Gentamicin (as control)			
	23.20 + 0.45 ^a^	24.10 + 0.45 ^a^	20.70 + 0.62 ^a^

K1, M1, and S1: unfermented samples; K2, M2, and S2: single culture using *Aspergillus niger*; K3, M3, and S3: co-cultured samples using *Aspergillus niger* and *Limosilactobacillus*; K4, M4, and S4: co-cultured samples using *Aspergillus niger* and *Lactiplantibacillus plantarum.*

## Data Availability

Data is contained within the article.
